# A house is not a home: a network model perspective on the dynamics between subjective quality of living conditions, social support, and mental health of refugees and asylum seekers

**DOI:** 10.1007/s00127-022-02419-3

**Published:** 2023-01-12

**Authors:** Laura Schilz, Solveig Kemna, Carine Karnouk, Kerem Böge, Nico Lindheimer, Lena Walther, Sara Mohamad, Amani Suboh, Alkomiet Hasan, Edgar Höhne, Tobias Banaschewski, Paul Plener, Michael Strupf, Erik Hahn, Malek Bajbouj

**Affiliations:** 1 Department of Psychiatry and Psychotherapy, Charité - Universitätsmedizin Berlin, Campus Benjamin Franklin, Berlin, Germany; 2 Department of Psychiatry, Psychotherapy and Psychosomatics, Medical Faculty, University of Augsburg, BKH Ausgburg, Augsburg, Germany; 3 Department of Child and Adolescent Psychiatry, Philipps-University Marburg, Marburg, Germany; 4 Department of Child and Adolescent Psychiatry and Psychotherapy, Central Institute of Mental Health, Medical Faculty Mannheim, University of Heidelberg, Mannheim, Germany; 5 Department of Child and Adolescent Psychiatry, Medical University Vienna, Vienna, Austria; 6 Department of Child and Adolescent Psychiatry and Psychotherapy, University of Ulm, Ulm, Germany; 7 Department of Psychiatry and Psychotherapy, University Hospital LMU, Munich, Germany

**Keywords:** Refugees, Housing, Living conditions, Social support, Post-migration, Depression, PTSD, Post-traumatic stress, Network analysis

## Abstract

**Background:**

Providing adequate living conditions for forcibly displaced people represents a significant challenge for host countries such as Germany. This study explores refugee mental health’s reciprocal, dynamic relationship with post-migration living conditions and social support.

**Methods:**

The study sample included 325 Arabic- or Farsi-speaking asylum seekers and refugees residing in Germany since 2014 and seeking mental health treatment. Associations between reported symptoms of post-traumatic stress and depression and the subjective quality of living conditions and perceived social support were analyzed using a two-level approach including multiple linear regression and network analyses.

**Results:**

Post-migration quality of living conditions and perceived social support were significantly associated with negative mental health outcomes on both levels. In the network, both post-migration factors were negatively connected with overlapping symptoms of psychiatric disorders, representing potential target symptoms for psychological treatment.

**Conclusion:**

Post-migration quality of living conditions and social support are important factors for refugee mental health and should be targeted by various actors fostering mental well-being and integration.

**Supplementary Information:**

The online version contains supplementary material available at 10.1007/s00127-022-02419-3.

## Introduction

Refugees and asylum seekers (RAS) constitute a particularly vulnerable group in terms of mental health, as they are exposed to multiple stressors before, during, and after their flight. Findings of a recent meta-analysis indicate substantially higher prevalence rates of depression, anxiety, and post-traumatic stress disorder (PTSD) in refugees resettling in high-income countries worldwide than among non-refugee populations [1]. These are consistent with findings of an epidemiological study reporting a higher prevalence of mental disorders among refugees in Germany compared to the host population [2]. Despite variation in estimates, prevalence rates range from 30.5 to 95.3% for any possible psychiatric disorder. High comorbidity rates between 64.2 and 94.2%, with PTSD and depression among the most common co-occurring disorders among refugees [3, 4], have been reported.

A growing body of literature emphasized the importance of taking pre-, peri-, and post-migration stressors and their impact on refugee mental health and well-being into account [5–7]. Post-migration stressors affecting mental health include delayed asylum procedures, the associated uncertainty, as well as the residence status. Moreover, a lack of social support and compromised housing conditions have been identified as crucial determinants of mental health [4, 8–10]. Research in low- and middle-income countries indicated that low living conditions in refugee camps were strongly associated with poor mental health outcomes [11, 12].

Yet, to date, there is a scarcity of empirical evidence investigating the effects of housing conditions on mental health for individuals resettling in Europe [13–17]. The majority of approximately 26.3 million refugees and 4.2 million asylum seekers worldwide were either displaced internally or have fled to neighboring, predominantly low- and middle-income countries in the Middle East [5, 18]. However, between 2014 and the first half of 2017, approximately 1.5 million individuals sought asylum in Germany, including 890,000 in 2015 alone [19, 20]. This posed unprecedented challenges to state and non-state actors on various levels, such as border control, asylum procedures, provision of housing, and physical and mental health care [3, 19, 21, 22].

Generally, the typical procedure after arrival in Germany foresees a short stay in emergency shelters or initial reception facilities with a subsequent transition to publicly provided refugee housing, concluding eventually with relocation to private accommodation at the latest after the termination of the asylum procedure [23]. However, multiple barriers, such as discrimination, lack of information, and required documents for the rental application process, language barriers, and potentially impaired cognitive resources due to psychological distress and mental health problems, obstruct the transition from temporary refugee housing to not only affordable, but also adequate private accommodation [24, 25]. Consequently, refugees often depend on support from others and social networks to find suitable housing conditions [26].

Collective refugee facilities in Germany tend to be associated with subjectively poorer quality of living conditions in comparison to private accommodation [27] and have been criticized for increased risk of developing psychosocial distress as a result of various limitations [28]. Living conditions in collective refugee facilities are more dissatisfying [27] with compromising factors such as the limited size of individual living space, noise, tensions, discrimination, lack of privacy and retreat opportunities, and perceived restrictions in autonomy and individuality (for an overview, see e.g., Gliemann and Szypulski [26]; Aumüller et al. [28]; Kreichauf [29]). Poor housing conditions can create an environment not conducive for studying and even resting—basic needs that are crucial for coping and processing traumatic experiences and integrational aspects of learning the new language [30]. Additionally, subjective perceptions of housing and environment quality may vary from objective situational conditions, sharing a bi-directional relationship to poor mental health or protective factors, such as social support. It is plausible that a good social network in place eases access to adequate housing in tense housing markets [26]. On the other hand, housing conditions might influence social relationships through mechanisms such as fostered interaction with people or isolation [28].

In a study in the UK, Campbell et al. [13] suggested a link between negative associations of accommodation satisfaction and emotional well-being with isolation mechanisms, as refugee accommodations were located in rather dispersed, often socioeconomic deprived social housing areas. In Germany, locations of collective refugee housing are often peripheral, partially isolated in industrial or commercial rather than residential areas [17]. Also, spatial demarcation with walls or fences can increase stigmatization and lack of social exchange with the host society [26]. Consistently, housing has been identified as a social determinant of integration [25].

Generally, lack of social exchange, isolation, and social withdrawal have been linked to reduced social support and experiences of social exclusion and are crucial stressors in the post-migration phase [7, 31]. Additionally, to isolate and lack of social exchange with the host society, many refugees experience separation from or loss of family members and friends [5, 32]. The family unit often plays an important role, particularly for individuals from Middle Eastern countries. Social support has been identified as a crucial protective factor concerning the mental health of refugees [7, 10, 33]. Other authors suggested a hindering effect of mental disorders such as PTSD or depression on the ability to maintain supportive social relationships [34, 35]. Taken together, lack of social support and poor living conditions represent two post-migration factors that share potentially reciprocal relationships with the mental health of refugees in the resettlement and acculturation phase.

The current study aims to fill gaps in the existing literature in two ways: In the first step of analysis, we investigate how the post-flight subjective quality of living conditions and social support influences mental health after accounting for pre- and peri-migration experiences on a higher order level of analysis. In a second step, on a lower order level of analysis, potentially reciprocal relationships are accounted for and explored by incorporating these contextual factors and symptoms of PTSD and depression in a shared network model using network analysis. This adds to an emerging body of research using this novel approach in the field of psychopathology [36, 37], and in particular with respect to PTSD [38, 39], PTSD in refugees [40], depression [41, 42], and comorbidity of PTSD and depression [39, 43, 44]. Finally, it contributes to the literature regarding comorbidity and refugee mental health using data from a large sample of asylum seekers and refugees seeking mental health treatment in Germany.

## Methods

### Study design

Data for this secondary analysis were collected as part of the Mental Health in Refugees and Asylum Seekers (MEHIRA; [22]) study, a nationwide multicentre randomized controlled trial in Germany involving eight study sites in Aachen, Marburg, Mannheim, Munich, Ulm, Tübingen, and two in Berlin. The study was approved by ethics committees at all participating sites and was registered in ClinicalTrails.gov (registration number: NCT03109028; registration date 11.04.2017) and adheres to the Declaration of Helsinki. Informed consent was obtained from all participants. A more detailed account of the study is described in the published study protocol [22].

### Participants and procedures

Participants were allocated to the different study sites through heterogeneous recruitment paths (general practitioners, social workers, etc.) via screenings. Potential participants were asked to complete the Refugee Health Screener-15 (RHS-15; [45]) and the depression scale from the Patient Health Questionnaire for adults and adolescents (PHQ-9, PHQ-A; [46, 47]) to assess clinically relevant depressive symptoms and psychological distress. Screening cut-offs for participation were defined as a score of $\ge 12$ for the items 1–14 or $\ge 5$ for item 15 in the RHS-15 and symptom presence marked as at least “several days” of five or more items on the PHQ-9. Further inclusion criteria of the MEHIRA study were (1) refugee or asylum seeker status as defined by the UNHCR, (2) native speaker of Arabic/Farsi and/or fluent in either English or German, and (3) age between 14 and 21 years for adolescents and between 18 and 65 years for adults. Psychotic symptoms, degenerative disorders, acute suicidality or missing informed consent were excluded from the study. For the present analysis, a cross-sectional sub-sample of baseline assessments between 2017 and 2019 was generated by selecting only participants who: (1) were between 18 and 65 years old, and (2) reported arrival year of 2014 or later. This led to a total number of $N = 351$ participants. Due to missing data, $n = 26$ participants were excluded from further analysis. The final sample consisted of $N = 325$ individuals.

### Measures

All measures were administered at baseline assessment in either Farsi or Arabic and were already available in the respective languages.

#### PHQ-9

Symptoms of depression were assessed using the nine-item module of the Patient Health Questionnaire (PHQ-9; [46, 48, 49]). Participants report the frequency of days with symptoms of a Major Depressive Episode (MDE) as described in the Diagnostic and Statistical Manual of Mental Disorders (DSM-5; [50]) on a 4-point Likert scale. Total scores range from 0 (no depression) to 27 (severe depression) with a commonly suggested cut-off score of 10 for a likely diagnosis of a depressive disorder [46] $\alpha = 0.75$).

#### HTQ

The Harvard Trauma Questionnaire (HTQ) is a widely used cross-cultural screening instrument to document exposure to potentially traumatic events (PTE) and trauma-related symptoms for use with refugee populations [51]. To assess symptoms of post-traumatic stress, participants were asked to rate 40 items (16 items covering PTSD symptoms according to DSM-IV [52] criteria plus 24 culture-specific items) on a 4-point Likert scale. Overall symptom severity of PTSD was measured using the mean score of all 40 items with a suggested cut-off score for a likely diagnosis of PTSD $\ge 2.5$ [51]. Part 1 of the HTQ was used to calculate the sum of reported PTE.

#### WHOQOL-BREF

The World Health Organization Quality of Life-BREF [53] is a brief self-rating tool assessing satisfaction with four core domains: (1) physical health, (2) psychological health, (3) social relationships, and (4) environment on a 5-point Likert scale. Overall internal validity was acceptable (Cronbach’s $\alpha =0.7$). Subjective quality of living conditions was assessed using the three items of living condition satisfaction, perceived safety, and healthy environment drawn from the environment domain. Perceived social support was measured using the sub-score of satisfaction with personal relationships, sex, and support from friends as facets of the social relationship domain. Cronbach’s alpha values for computed sub-scores in the present sample ranged from $0.60$ (for social support) to $0.65$ (for living conditions). Based on only three items for each subgroup, these should be interpreted with caution [54].

### Data analysis

Data preparation, descriptive statistics, and multiple linear regression analysis were conducted in SPSS IBM Version 25. Network analysis was done using the statistical software of *R* (Version 3.6.1 [55]). Missing data were present on 4.34% of the items. Cases with more than 20% missing data were omitted from analyses ($n=26$). Additional missing data were handled using the listwise deletion method in multiple linear regression analyses and the built-in pairwise deletion function in the *R* package *qgraph* [56] for network analyses.

#### Multiple linear regression

Separate multiple linear regression analyses were conducted using block-wise entering of variables, potentially predicting the symptom severity of PTSD and depression. Control variables comprised variables for time since arrival as the number of months, loss of social status as comparison of subjectively perceived social status as of today and before migration as well as dummy variables for permanent residence permit versus temporary/no residence permit, for relationship status and for living with or without a partner.

#### Network analysis

Network analysis was performed in four steps according to a recent tutorial by Epskamp and Fried [57]: (1) network model estimation, (2) model metrics, (3) identification of bridges across communities, and (4) post hoc accuracy and stability analysis using the R packages *bootnet, qgraph* and *glasso* [56, 58, 59]. Forty items were included, of which 19 were combined to form seven composite variables including those theorized to tap the same symptom (e.g., concentration problems) to reduce possible topological overlap [60]. In the present study, a total of 26 nodes consisted of symptoms of depression and post-traumatic stress, and one node each for subjectively perceived quality of living conditions and social support.

A more detailed description of the different methodological steps and visualization basics can be found in supplementary materials. Epskamp and Fried [57] comprehensively explain network estimation and interpretation. The network model estimation resulted in a regularized network graphically depicted with a colorblind-sensitive theme using a modified Fruchterman–Reingold [61] algorithm according to cluster grouping based on the DSM-5 factor model of PTSD and the two-factor model of depression supported by, e.g., Elhai et al. [62].

A centrality index of node strength was calculated for assessing the relevance of nodes [63–65]. According to the network approach to psychopathology, the so-called bridge symptoms may be crucial in the development and maintenance of comorbidity [66]. The inference measure of bridge strength to identify bridge symptoms was computed using the *R* package *networktools* [67, 68].

Finally, using the *R* package *bootnet*, bootstrap routines with 2500 iterations were used to assess (1) accuracy of network connections computing 95% confidence intervals around estimated regularized edge weights and centrality indices, and (2) stability of centrality and bridge strength parameters calculating a correlation stability coefficient (CS coefficient) that should not be below 0.25 and is preferably above 0.50 [58].

## Results

### Descriptive statistics

The final sample included $N=325$ individuals, comprising 100 female (30.8%) and 225 male participants (69.2%) after missing data handling. Mean score for PTSD symptom severity reported in the HTQ was $M = 2.59$ (SD = 0.58, range: 1.00–3.83) and $M = 17.18$ (SD = 5.29, range: 5.00–27.00) in the PHQ-9 for depression (see Table A1 in the supplementary material for descriptive statistics and Table A2 for mean scores). Criteria for a likely diagnosis of both depression and PTSD were met by 183 participants (56%), by 119 (36%) for one diagnostic category, and by 23 (7%) for none (see Table A3 in supplementary materials).

Sociodemographic characteristics of the study sub-sample are presented in Table A1 in the supplementary materials.

### Multiple linear regression analyses

Multiple linear regression analyses were performed to examine the influence of socio-demographic and migration-related variables on symptoms of PTSD and depression with specific regard to post-migration living conditions and social support. The addition of cumulative potentially traumatic events (PTE) to the base model significantly improved the model, with 18.9% of explained variability in PTSD symptom severity in Model 1a and 13.8% for depression in model 2b, compared to 6.4% and 8.5% in model 1a and model 1b, respectively (see Table 1 for PTSD and Table 2 for depression). Entering social support and living conditions constituted a significant improvement in model fit (PTSD: $F(2, 272) = 34.63$, $p<0.001$; depression: $F(2, 272) = 14.58$, $p<0.001$).

The inclusion of these variables explained more additional variance than entering PTE. The adjusted $R^{2}$ in the final model was 48.8% for PTSD and 38.4% for depression. The increase in $R^{2}$ and explained variability were higher in the model with PTSD than with depression.

In the full model predicting PTSD (Model 4a), higher symptom severity in the sample was significantly associated with poorer living conditions ($$\beta = -\,0.18$$, $$t =-\,3.90$$, $$p<0.001)$$ and lower social support ($$\beta = -\,0.18$$, $$t =-\,3.58$$, $$p<0.001$$). Further significant associations were found for female gender ($$\beta = -\,0.15$$, $$t =-\,2.86$$, $$p = 0.005$$), less years of schooling ($$\beta = -\,0.10$$, $$t =-\,2.09$$, $$p = 0.04)$$, higher number of PTE ($$\beta = 0.21$$, $$t =4.59$$, $$p<0.001)$$, and higher depression symptom severity ($$\beta = 0.43$$, $$t =8.68$$, $$p<0.001$$).

Stronger symptom severity of depression was significantly associated with poorer living conditions ($$\beta = -\,0.12$$, $$t =-\,2.10$$, $$p = 0.037)$$ and lower social support ($$\beta = -\,0.24$$, $$t =-\,3.98$$, $$p<0.001)$$ in Model 3b. Further significant predictors in this stage were perceived loss of social status ($$\beta = 0.15$$, $$t =2.76$$, $$p = 0.006)$$, increased cumulative PTE ($$\beta = 0.19, t =3.38$$, $$p = 0.001)$$, and female gender ($$\beta = -\,0.18$$, $$t =-\,2.82$$, $$p = 0.005$$). With the entering of the significantly related variable of higher symptom severity of PTSD ($$\beta = 0.51$$, $$t =8.68$$, $$p<0.001)$$ into Model 4b, only perceived loss of social status remained significant ($$\beta = 0.14$$, $$t =2.83$$, $$p = 0.005$$).

**Table 1:** Results of multiple linear regression predicting post-traumatic stress symptom severity

		Model 1a			Model 2a			Model 3a			Model 4a		
		*B*	SE	$\beta$	*B*	SE	$\beta$	*B*	SE	$\beta$	*B*	SE	$\beta$
(1)	Gender$$^{\text {a}}$$	$$-\,0.27$$	0.08	$$-\,0.21^{***}$$	$$-\,0.34$$	0.08	$$-\,0.27$$	$$-\,0.28$$	0.07	$$-\,0.22^{***}$$	$$-\,0.19$$	0.06	$$-\,0.15$$
	Age	0.01	0	0.11	0	0	0.09	0	0	0.03	0	0	0.01
	Years of schooling	$$-\,0.02$$	0.01	$$-\,0.12$$	$$-\,0.01$$	0.01	$$-\,0.07$$	$$-\,0.02$$	0.01	$$-\,0.13^{**}$$	$$-\,0.01$$	0.01	$$-\,0.1$$
	Time since arrival	0	0	$-\,0.06$	0	0	$-\,0.05$	0	0	$-\,0.07$	0	0	$-\,0.07$
	Married w/o partner$^{\text {b}}$	0.23	0.14	0.1	0.18	0.13	0.08	0.13	0.12	0.06	0.03	0.11	0.01
	Single$^{\text {a}}$	0.18	0.1	0.15	0.14	0.09	0.12	0.08	0.08	0.07	0.08	0.07	0.07
	Div./Sep./Wid.$^{\text {b}}$	0.11	0.21	0.03	0.11	0.19	0.03	0.07	0.17	0.02	0.08	0.15	0.02
	Permanent residence permit$^{\text {c}}$	$-\,0.23$	0.19	$-\,0.07$	$-\,0.06$	0.18	$-\,0.02$	$-\,0.06$	0.16	$-\,0.02$	$-\,0.08$	0.14	$-\,0.02$
	Perceiv. loss social status $$^{\text {d}}$$	0.17	0.07	0.15$$^{**}$$	0.11	0.07	0.09	0.03	0.06	0.03	$$-\,0.04$$	0.05	$$-\,0.04$$
(2)	PTE				0.03	0	0.37$$^{***}$$	0.02	0	0.29$$^{***}$$	0.02	0	0.21$$^{***}$$
(3)	Living conditions							$$-\,0.14$$	0.03	$$-\,0.23$$	$$-\,0.11$$	0.03	$$-\,0.18^{***}$$
	Social support							$$-\,0.16$$	0.03	$$-\,0.28$$	$$-\,0.1$$	0.03	$$-\,0.18^{***}$$
(4)	Depression sympt. severity										0.05	0.01	0.43$$^{***}$$
	$$\Delta R^{2}$$						0.123$$^{***}$$			0.159$$^{***}$$			0.136$$^{***}$$
	Adj. $R^{2}$			0.064			0.188			0.348			0.488

*Married w/o partner* married and living without partner, *Div./Sep./Wid.* divorced/separated/widowed, *PTE* cumulative potentially traumatic events score

**p < 0.05. ***p < 0.01 for coefficients as well as model comparison

Reference categories of dummy variables: $^{\text {a}}$Female, $^{\text {b}}$Married and living with partner, $^{\text {c}}$Temporary/no residence permit, $^{\text {d}}$No perceived loss of social status

**Table 2:** Results of multiple linear regression predicting depression symptom severity

		Model 1b			Model 2b			Model 3b			Model 4b		
		*B*	SE	$\beta$	*B*	SE	$\beta$	*B*	SE	$\beta$	*B*	SE	$\beta$
(1)	Gender$$^{\text {a}}$$	$$-\,2.02$$	0.77	$$-\,0.17^{***}$$	$$-\,2.47$$	0.76	$$-\,0.21***$$	$$-\,2.05$$	0.72	$$-\,0.18^{***}$$	$$-\,0.73$$	0.66	$$-\,0.06$$
	Age	0.05	0.04	0.11	0.05	0.04	0.10	0.02	0.04	0.05	0.02	0.03	0.03
	Years of schooling	$-\,0.08$	0.08	$-\,0.06$	$-\,0.04$	0.07	$-\,0.03$	$-\,0.10$	0.07	$-\,0.08$	$-\,0.02$	0.06	$-\,0.01$
	Time since arrival	0.00	0.02	0.01	0.01	0.02	0.02	0.00	0.02	0.00	0.01	0.02	0.04
	Married w/o partner$$^{\text {b}}$$	2.74	1.29	0.13$$^{**}$$	2.39	1.26	0.11	2.03	1.20	0.10	1.43	1.07	0.07
	Single$^{\text {b}}$	0.72	0.86	0.07	0.49	0.84	0.05	0.08	0.81	0.01	$-\,0.29$	0.71	$-\,0.03$
	Div./Sep./Wid.$^{\text {b}}$	0.02	1.89	0.00	- 0.01	1.83	0.00	$-\,0.24$	1.75	$-\,0.01$	$-\,0.59$	1.55	$-\,0.02$
	Permanent residence permit$^{\text {c}}$	$-\,0.55$	1.74	$-\,0.02$	0.46	1.70	0.02	0.31	1.63	0.01	0.60	1.45	0.02
	Perceiv. loss social status$$^{\text {d}}$$	2.57	0.63	0.24$$^{***}$$	2.17	0.62	0.20$$^{***}$$	1.65	0.60	0.15$$^{***}$$	1.50	0.53	0.14$$^{***}$$
(2)	PTE				0.19	0.05	0.25$$^{***}$$	0.15	0.04	0.19$$^{***}$$	0.03	0.04	0.04
(3)	Living conditions							$$-\,0.66$$	0.32	$$-\,0.12^{**}$$	- 0.01	0.29	0.00
	Social support							$$-\,1.26$$	0.32	$$-\,0.24^{***}$$	- 0.50	0.29	- 0.10
(4)	PTSD sympt. severity										4.69	0.54	0.51$$^{***}$$
	$\Delta R^{2}$						0.054***			0.081***			0.164***
	Adj. $R^{2}$			0.085			0.138			0.216			0.384

*Married w/o partner* married and living without partner, *Div./Sep./Wid.* divorced/separated/widowed, *PTE* cumulative potentially traumatic events score

**p < 0.05. ***p < 0.01 for coefficients as well as model comparison

Reference categories of dummy variables: $^{\text {a}}$Female, $^{\text {b}}$Married and living with partner, $^{\text {c}}$Temporary/no residence permit, $^{\text {d}}$No perceived loss of social status

### Network of symptoms of depression and post-traumatic stress with social support and living conditions

**Fig. 1:**
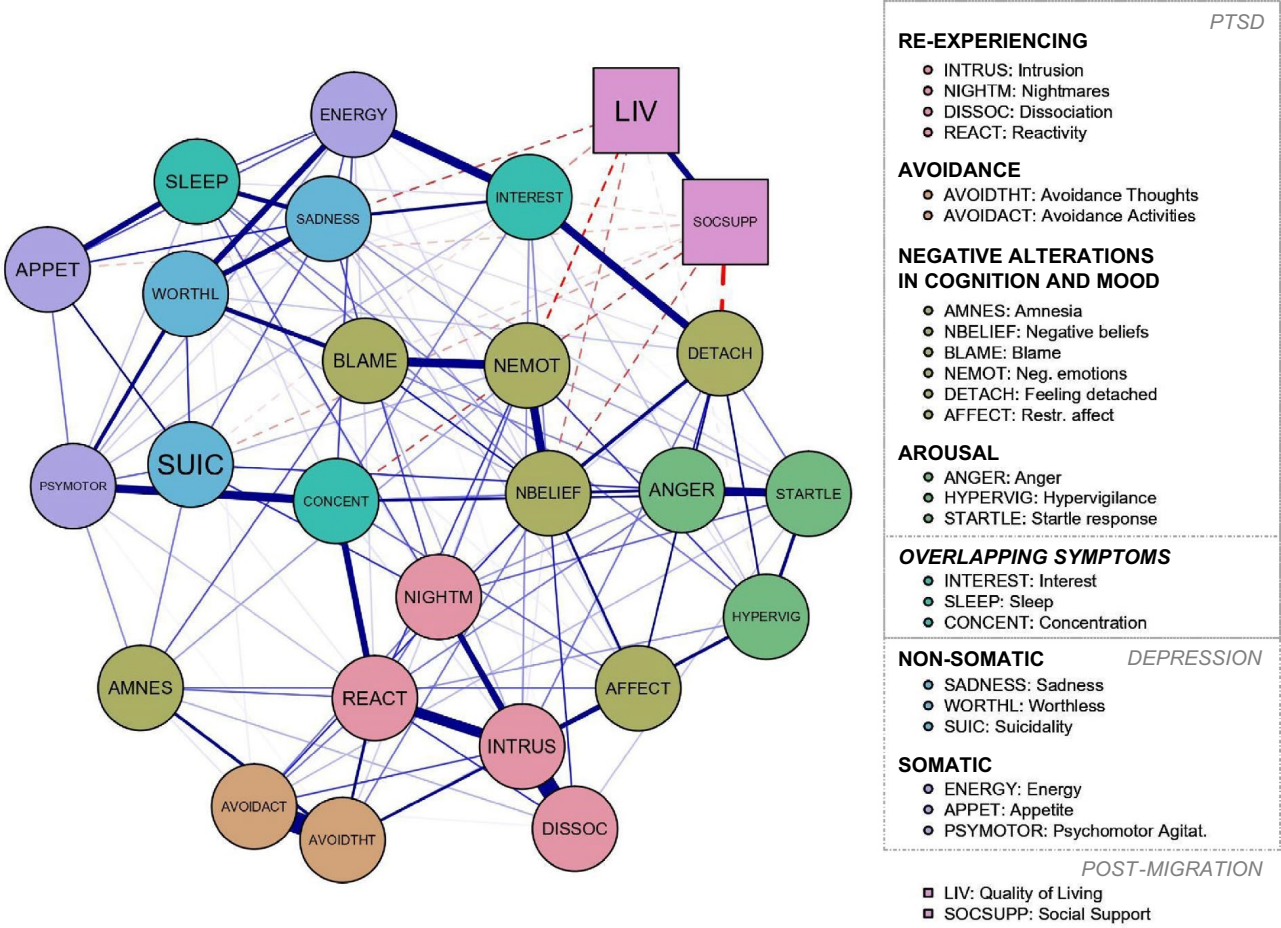
Partially regularized network estimated using symptom nodes of mental health outcomes (round shapes) and non-symptoms, post-migration factor nodes (squares). Colours correspond to the DSM-5 factor model of PTSD groups, overlapping symptoms of depression and PTSD, and the two-factor model of depression. Thicker edges represent stronger connections; solid, blue edges depict positive associations; dashed, red edges characterise negative associations

Figure 1 shows a graphical representation of the estimated regularized correlation network structure.

Nodes representing psychological symptoms of depression and PTSD were positively interconnected. Figure A1 in the supplementary material presents the resulting plot of non-parametric bootstrap testing on edge-weight accuracy with several overlapping confidence intervals around estimated edge weights.

The post-migration factor nodes of subjectively perceived living conditions and social support revealed strong positive interconnections. Both non-symptom nodes were negatively connected to symptom nodes, in particular to those of high centrality or bridge functions. Six of the symptom nodes evidence edges with living conditions, and seven with social support. These were all negatively associated (see also the flow diagrams in Figure A2 and A3 in supplementary material). Connections were particularly strong for social support and feelings of detachment and living conditions, and negative trauma-related emotions. For these connections, non-parametric bootstrapping revealed significantly differing edge weights in comparison to nearly all other edge weights (see Figure A4 in the supplement for results of bootstrap testing for significant differences between edge weights).

The network centrality indices of edge strength were highest for negative beliefs, intrusion, reactivity, feeling detached, and negative emotional state, and lowest for the nodes of quality of living, amnesia as a symptom of PTSD, and appetite as a symptom of depression. Centrality indices are depicted in Figure A5 in the supplements; Figure A6 shows robustness test results of symptom centrality under sub-setting. Bootstrap routines assessing the CS coefficients were above 0.2 (CS = 0.44). Results of non-parametric bootstrap testing revealed only a few nodes with significant differences in centrality (e.g., negative beliefs to quality of living). In contrast, nodes with the highest and lowest centrality values mostly did not differ significantly among one another (see Figure A7 in the supplementary material).

**Fig. 2:**
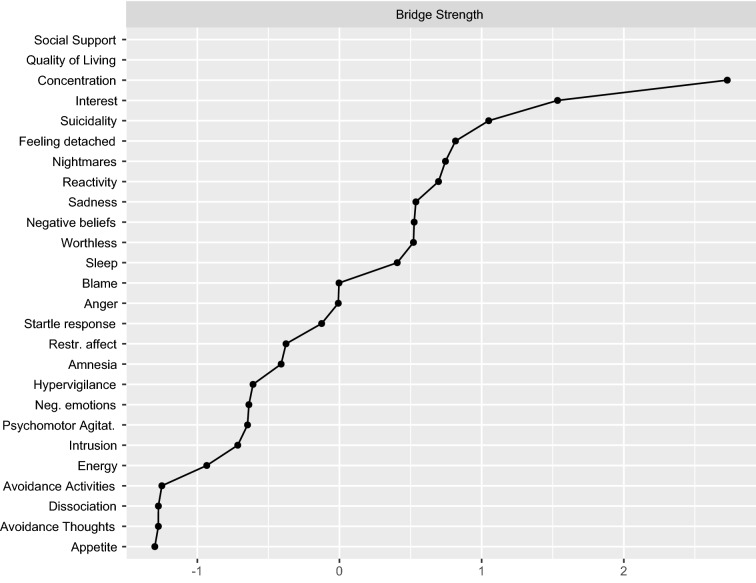
Bridge strength plotted using standardized *z* scores. Conceptually predefined communities for identifying bridge symptoms in between were depression and post-traumatic stress symptoms groups. Social support and quality of living were excluded

Finally, the symptom nodes’ concentration and interest, as well as suicidality, detachment, and nightmares, were the most central bridging nodes between the depression and PTSD symptom groups (see Fig. 2).

## Discussion

This study investigated the potentially reciprocal association of symptoms of post-traumatic stress and depression symptoms with post-migration subjective quality of living conditions and perceived social support in a sample of treatment-seeking RAS that arrived in Germany after 2014.

### Subjectively perceived living conditions and mental health outcomes

On a scale level, findings indicate a negative relationship between subjectively perceived living conditions and mental health outcomes after controlling for socio-demographic and migration-related factors. Both post-migration factors explained more variance in symptom severity than the experience of potentially traumatic events before and during migration. This adds to relatively scarce literature addressing the impact of living conditions and environment on mental health and well-being of refugees in high-income resettlement countries [24]. Leiler et al. [14] reported high levels of psychological distress and low subjective quality of life in refugee housing facilities in Sweden, whereas refugees in the Netherlands reported negative impacts of worries about the housing situation on their mental well-being [16]. In contrast, Georgiadou et al. [3] found no association between depression and PTSD with the type of accommodation in Germany, while Walther et al. [69] reported differences in psychological distress of refugees arriving in Germany after 2013 depending on whether they lived in a collective accommodations compared to a shared or own apartment. In addition, results of a study by Nutsch et al. [15] in Germany indicated a protective effect of satisfaction with living conditions on depression.

In the present study, living conditions were measured, including satisfaction with housing conditions along with a sense of living in safety and a healthy environment.

### Perceived social support and mental health outcomes

Social support has been repeatedly identified to play a crucial role in developing and maintaining mental health problems in the previous studies [10, 33, 70]. In the current study, increased levels of depression and PTSD symptom severity were associated with lower levels of perceived social support after controlling for post- and pre-settlement factors. This contributes to a meta-analysis study where lack of social support was identified as a crucial predictor for depression even 5 years or more after resettlement [6]. Other authors have highlighted the importance of traumatized persons’ ability to communicate about their experiences, the so-called disclosure, and recognition as victims by their surroundings [70].

### Network model of living conditions, social support, and co-occurring symptoms of depression and PTSD

Results from network analyses suggest unique pathways and mechanisms in the reciprocity between living conditions and social support and co-occurring depression and PTSD. This adds to pre-existing literature of depression and PTSD symptoms in a network [43, 44]. Similar to the present study, results from network analyses studying PTSD indicated centrality of nodes, such as detachment [39, 71], and concentration as central and bridge symptom in network analyses of single or comorbid disorders [72, 73].

Negative values and varying weights of the edges connecting the two non-symptom nodes with symptom nodes are consistent with the previously described findings of the regression analyses. Moreover, in the full model of regression analyses predicting depression living conditions and social support were no longer significant after controlling for PTSD. This is in line with reduced living conditions and social support connections to depression nodes in the estimated network when simultaneously including PTSD symptoms and potentially reflecting mediation processes [57].

In the estimated network, social support and living conditions shared no connection to any nodes of the PTSD factors of re-experiencing and avoidance clustered according to the DSM five-factor model. This is consistent with previous research [33], highlighting significantly weaker social support relations to the factors of re-experiencing and avoidance.

Several authors suggest the existence of trauma-specific factors and found strong associations of the PTSD cluster factor of negative alterations in cognition and mood with the non-somatic, affective factor of depression [33, 74].

In the present study, both post-migration factor nodes were connected to negative beliefs. The nodes with the highest centrality strength were symptoms of negative beliefs, feeling detached, and negative trauma-related emotions. Consistent with previous research [39], non-symptom nodes did not yield high centrality strengths but were sharing stronger relationships with particularly central nodes. Central symptoms may potentially be considered as targets for treatment [75] and interventions, as well as “external” factors sharing unique pathways with central symptoms. In fact, negative trauma-related cognitions have been subject to change during treatment for PTSD and associated with symptom reduction [76].

Finally, according to the network theory of psychopathology, bridge nodes are considered crucial in the etiology and/or maintenance of comorbidity of disorders [66, 67]. Living conditions and social support were connected to suicidal ideation, interest, concentration, and sadness nodes, and these have been identified as major bridge nodes in the present network. In the previous network analyses studying depression as a comorbid disorder, concentration problems and sadness also constituted the central or bridge symptoms [67, 68]. Comorbidity is associated with a worse course of illness and prognosis of treatment response as well as with poor quality of life [67, 77]. Hence, further exploration of symptoms bridging these disorders—and potential external interacting factors might be promising.

### Limitations and future directions

Regarding limitations of the findings, stability and accuracy may have been compromised by the limited sample size with respect to the generally high number of parameters to be estimated in network analyses. Confidence levels were largely overlapping, and findings of the bootstrap analyses indicate a cautious interpretation of estimated metrics. Validity and reliability might be impaired due to only acceptable internal consistency of some scales as well as translated versions of questionnaires.

Symptom nodes were assessed using pre-existing measures initially constructed under the assumptions of an underlying latent factor and several items were aggregated due to power reasons. In addition, only subjectively assessed living conditions were included.

Next, the outcomes presented here are secondary analyses of the MEHIRA sample and the sample size may be underpowered. Thus, the findings are exploratory and future studies with larger sample sizes could include items developed for network analysis and culture-specific symptoms.

Furthermore, generalizability is compromised due to cross-sectional data and sample selection. The latter was based on criteria that depended on the variables included in the analyses, leading to limitations regarding Berkson Bias [78]. However, from an ecological validity perspective, the sample selection, without differentiation by country of origin but with Arabic- or Farsi-speaking and majority male participants, is potentially representative of those seeking treatment in the current period: The majority of refugees arriving in Germany since 2014 were predominantly male [20], and about two-thirds were mainly from the Syrian Arab Republic, Afghanistan, Iraq, and the Islamic Republic of Iran [79]. Another limitation to generalizability could be that the data were collected exclusively in Germany, which is only one of the high-income countries that are heterogeneous in terms of migration policies. Future studies in longitudinal and possibly cross-national designs may allow for better generalizability, inferences about directionality, or temporal predictability. Recent tendencies in network analysis research target dynamic, temporal, and individual network structures [80, 81], representing a promising therapy research approach.

Both post-migration factors may be crucial in the acculturation phase of refugees. Positive effects on mental health and facilitation of social integration might be achieved through policies and interventions: For instance, these could aim at strengthening social networks and relationships, reducing barriers and discrimination regarding the private rental market transition, and providing accommodation in socially and culturally diverse areas along with “normal” housing to prevent discrimination and social isolation [5, 25, 82, 83]. This may also contribute to the valuable considerations of White and Van der Boor [84] that discuss, for instance, the possibility of increasing the effectiveness of psychological interventions for some forcibly displaced people (FDP) by simultaneously providing positive social interactions. They propose the use of the capability approach (CA) as a framework to understand the impact of displacement-related stressors on FDP. Following this approach, lower social support and poorer living conditions could represent barriers to RAS ability to engage in valued forms of well-being and functioning. Conversely, other opportunities and barriers could also influence subjective evaluations and perceptions of current social support and quality of life and the extent of their impact on mental health.

## Conclusion

This study explored two aspects of post-migration factors, subjectively perceived living conditions and social support. Network analyses revealed that living conditions and social support exhibit unique negative associations with psychological symptoms reported by treatment-seeking RAS. They can be considered social determinants of integration and predictors of refugee mental health that may positively or negatively interfere with treatment. Therefore, various actors should target them on different levels, such as being subject to policies and psychosocial interventions. Whereas host countries may initially focus on border control, provision of basic care, and primary accommodation; in the long term, the challenges go far beyond the need of having a roof over one’s head. Post-migration socioeconomic and interpersonal factors, such as the quality of living conditions, opportunities for social exchange, and perceived social and governmental support, interact not only reciprocally with refugees’ mental health, but also with the chance of successful integration into society over time.

## Supplementary Information

Below is the link to the electronic supplementary material.

Supplementary file 1 (pdf 498 KB)

Supplementary file 2 (pdf 66 KB)

Supplementary file 3 (pdf 65 KB)

Supplementary file 4 (pdf 98 KB)

Supplementary file 5 (pdf 23 KB)

Supplementary file 6 (pdf 22 KB)

Supplementary file 7 (pdf 29 KB)

## Declarations

### Code and data availability

The raw data supporting the conclusions of this manuscript will be made available by the authors, without undue reservation, to any qualified researcher.
