# Supplementary material for: A house is not a home: a network model perspective on the dynamics between subjective quality of living conditions, social support, and mental health of refugees and asylum seekers
Source: Soc Psychiatry Psychiatr Epidemiol. 2023 Jan 12;58(5):757–68. doi: 10.1007/s00127-022-02419-3 (PMC10097787; doi:10.1007/s00127-022-02419-3)
Supplement: Supplementary file 1 — (pdf 498 KB) [file 127_2022_2419_MOESM1_ESM.pdf]

Appendix A

Table A1 Sociodemographic and migration-related characteristics of participants

	n	%	*M (SD)*	range	*N*
Gender					325
Female	100	30.8			
Male	225	69.2			
Age (years)			30.61 (10.63)	18 - 65	325
18 - 25	134	41.23			
26 - 35	92	28.31			
36 - 45	64	19.69			
46 - 55	30	9.23			
56 - 65	5	1.54			
Relationship status					323
Single	164	50.8			
Married and living with partner	91	28.2			
Married and without partner	27	8.4			
Sep./Div./Wid.	41	12.7			
Years of schooling			8.53 (4.11)	0 - 13	307
PTE			12.01 (6.83)	0 - 34	325
Residence permit					323
Permanent	13	4.0			
Temporary	282	87.3			
No legal	26	8.0			
Other	2	0.6			
Time since arrival (in months)			33.34 (14.07)	0 - 61	325
Social status loss					315
No	121	38.4			
Yes	194	61.6			
Living situation					324
Own/shared apartment	154	47.5			
Refugee housing	170	52.5			

Table A2 Descriptive statistics of variables

Variable	Mean	Standard Deviation	Min	Max	*N*
Depression symptom severity	17.18	5.29	5.00	27.00	325
PTSD symptom severity	2.59	0.58	1.00	3.83	325
Quality of living conditions	2.93	0.97	1.00	5.00	318
Social support	2.94	1.00	1.00	5.00	314
Intrusion	1.90	0.98	0.00	3.00	321
Nightmares	1.71	1.08	0.00	3.00	319
Dissociation	1.58	1.08	0.00	3.00	322
Reactivity	1.90	1.03	0.00	3.00	323
Avoidance thoughts	1.70	1.06	0.00	3.00	321
Avoidance activities	1.77	1.12	0.00	3.00	320
Amnesia	1.24	1.12	0.00	3.00	319
Negative beliefs	2.66	0.65	0.00	3.00	325
Blame	1.68	1.12	0.00	3.00	320
Negative emotions	1.81	1.13	0.00	3.00	321
Feeling detached	1.71	1.12	0.00	3.00	322
Restricted affect	1.38	1.07	0.00	3.00	323
Anger	1.91	1.01	0.00	3.00	324
Hypervigilance	1.55	1.10	0.00	3.00	311
Exaggerated startle response	1.67	1.06	0.00	3.00	322
Interest	2.26	0.84	0.00	3.00	325
Sleep	2.46	0.84	0.00	3.00	325
Concentration	2.48	0.78	0.00	3.00	325
Sadness	2.33	0.82	0.00	3.00	323
Energy	2.19	0.92	0.00	3.00	323
Appetite	1.75	1.15	0.00	3.00	324
Worthless	1.88	1.07	0.00	3.00	323
Psychomotor agitation	1.56	1.11	0.00	3.00	323
Suicidality	1.16	1.15	0.00	3.00	325

Table A3 Likely depression and PTSD diagnoses

		Female (n = 100)		Male (n = 225)		Total (N = 325)	
No diagnosis	PHQ-9 $<$ 10 & HTQ $<$ 2.5	4	4	19	8	23	7
Depression	PHQ-9 $\geq$ 10	23	23	89	40	112	34
PTSD	HTQ $\geq$ 2.5	3	3	4	2	7	2
Comorbidity	PHQ-9 $\geq$ 10 & HTQ $\geq$ 2.5	70	70	113	50	183	56

Note. PHQ-9 = Patient Health Questionnaire-9; HTQ = Harvard Trauma Questionnaire. A score on the PHQ-9 $\geq$ 10 indicates the presence of moderate to severe depression. A score on the HTQ $\geq$ 2.5 indicates the likely presence of PTSD.

Network model estimation

A correlation matrix was estimated using pairwise complete observations and the *cor_auto* function within the *bootnet* package [?]. Resulting non-zero partial correlation coefficients were drawn graphically as weighted and positive or negative edges between nodes. Regularized estimation methods using a graphical least absolute shrinkage and selection operator (GLASSO) procedure with Extended Bayesian Information Criterion (EBIC) selection [?] were applied by means of the R package *qgraph* [?] within the *bootnet* package [?] to calculate a network of reduced complexity that were manageable for interpretation.

Flow diagrams

Unique associations of interest linking quality of living conditions and social support were depicted by flow diagrams using the *flow* function within *qgraph* [?].

Centrality index

A centrality index of node strength indicates a node's absolute sum of connectivity with all other nodes. It has recently been suggested as a more reliable centrality index concerning the importance of nodes, particularly for psychological data [? ? ?]. A more extensive overview of centrality indices and their interpretation is presented elsewhere [? ? ?].

Bridge symptoms

The inference measure of bridge strength assesses the total connectivity (as the sum of absolute edge values) of a node to all other nodes of conceptually predefined communities of PTSD, depression symptoms groups and eventually the post-migration variables group.

CS coefficient

A CS coefficient indicates with a 95% certainty the maximum proportion of cases droppable from the sample while retaining a correlation of .70 with estimated centrality coefficients. According to [?], it should not be below .25 and is preferably above .50.

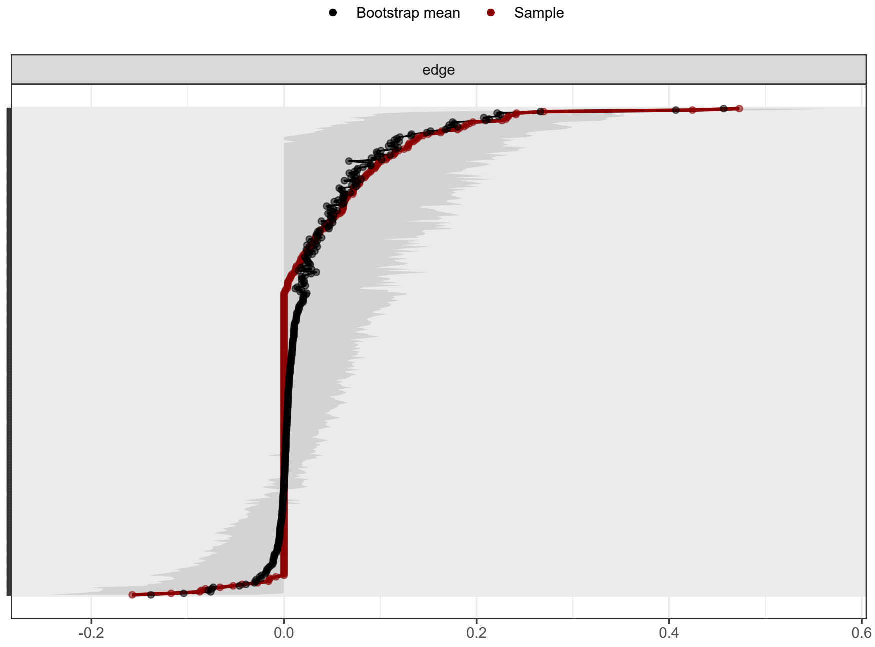

Fig. A1 Bootstrapped 95% confidence intervals of all estimated edge-weights. The red line indicates the sample values, the black line values from the bootstrap estimation, values are ordered from the lowest to the highest edge-weights. The y-axis labels have been omitted to avoid overlapping descriptions. Each horizontal line constitutes one edge in the estimated network.

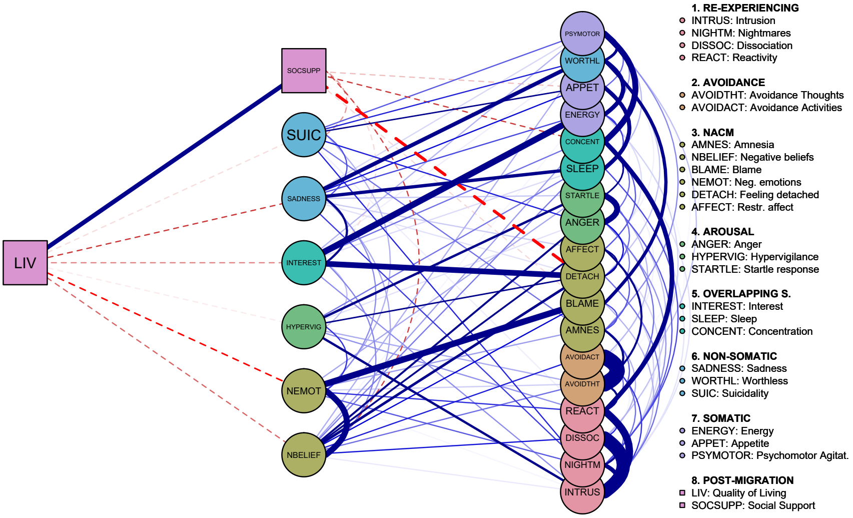

Fig. A2 Network flow diagram for quality of living conditions depicting the partially regularized network paths that connect the node of quality of living with all other nodes in the network. Non-symptoms, post-migration factor nodes are in the form of squares. Symptom nodes are grouped in potential factors of depression, PTSD and overlapping symptoms of both. Thicker edges represent stronger connections, solid, blue edges depict positive associations, negative associations are depicted by dashed, red edges.

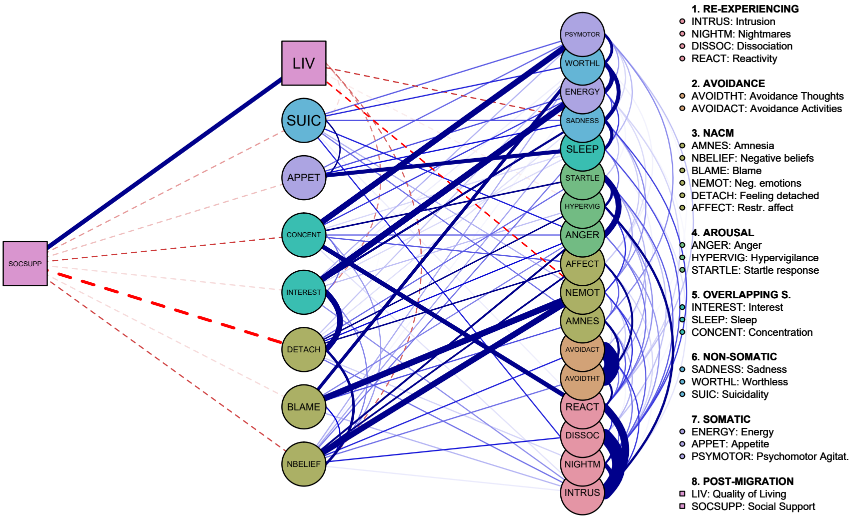

Fig. A3 Network flow diagram for social support depicting the partially regularized network paths that connect the node of social support with all other nodes in the network. Non-symptoms, post-migration factor nodes are in the form of squares. Symptom nodes are grouped in potential factors of depression, PTSD and overlapping symptoms of both. Thicker edges represent stronger connections, solid, blue edges depict positive associations, negative associations are depicted by dashed, red edges.

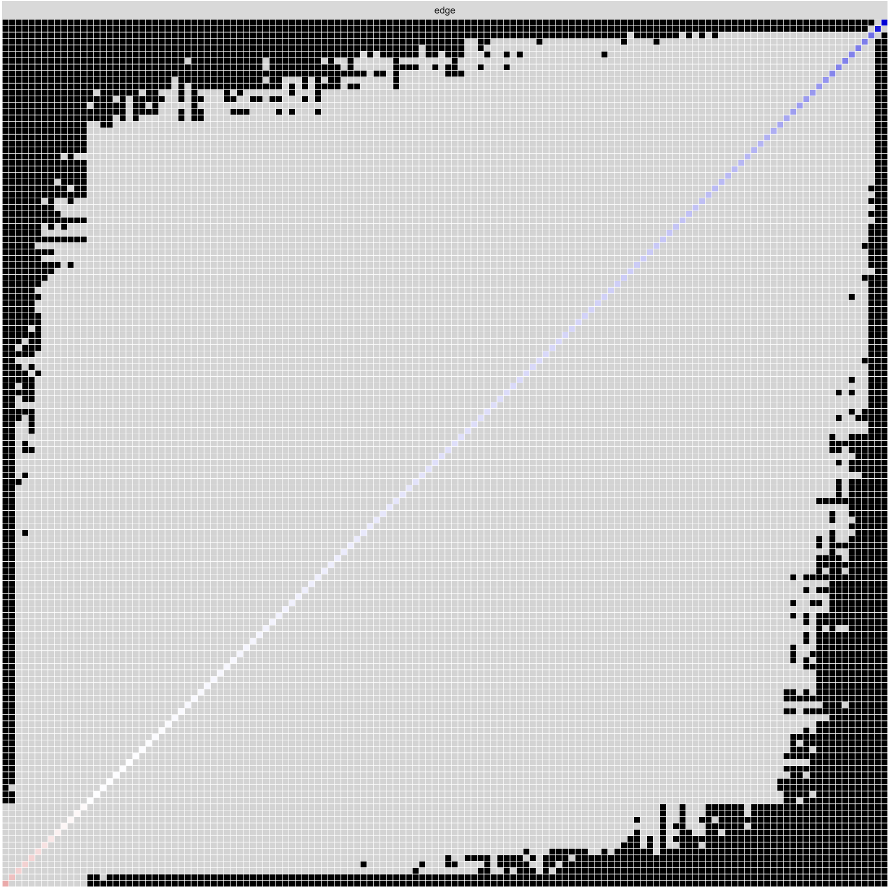

Fig. A4 Significant differences in edge weights: Results of bootstrapped difference tests ($\alpha = .05$) between non-zero edge weights of the 26 nodes with 2500 iterations. Black boxes indicate edges with significant differences, gray boxes indicate edges not differing significantly from one-another. Red and blue colors in the diagonal boxes indicate positive (blue) or negative (red) associations.

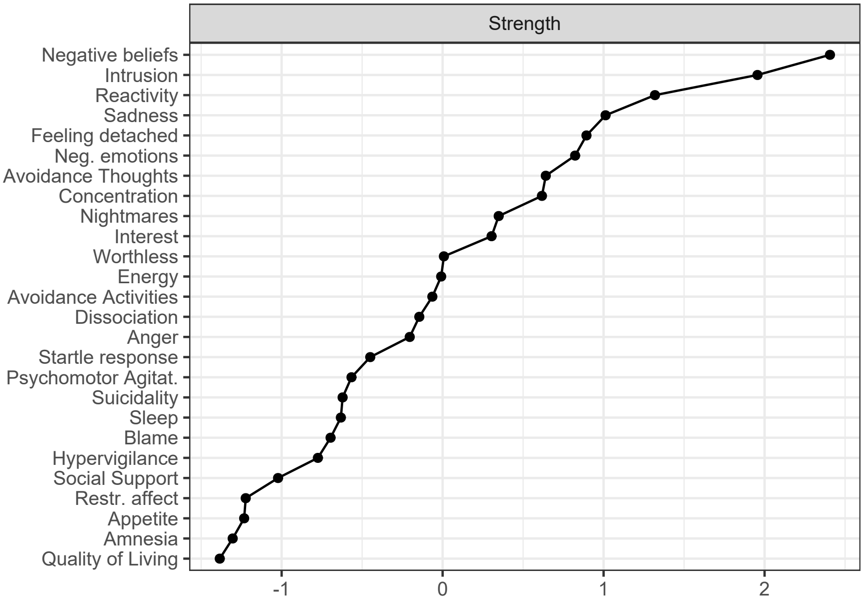

Fig. A5 Network centrality indices of edge strength plotted using standardized z-scores.

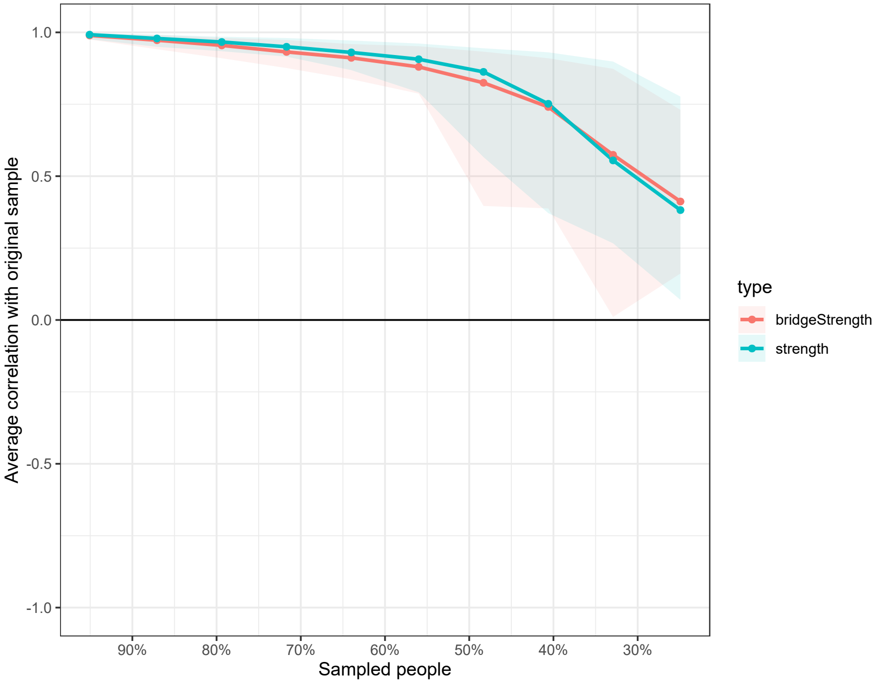

Fig. A6 Robustness of symptom centrality under sub-setting: Average correlations between centrality indices of a bootstrapped case-dropped sample and the original sample. Lines represent the means and colored areas indicate the $2.5th - 97.5th$ quantile range.

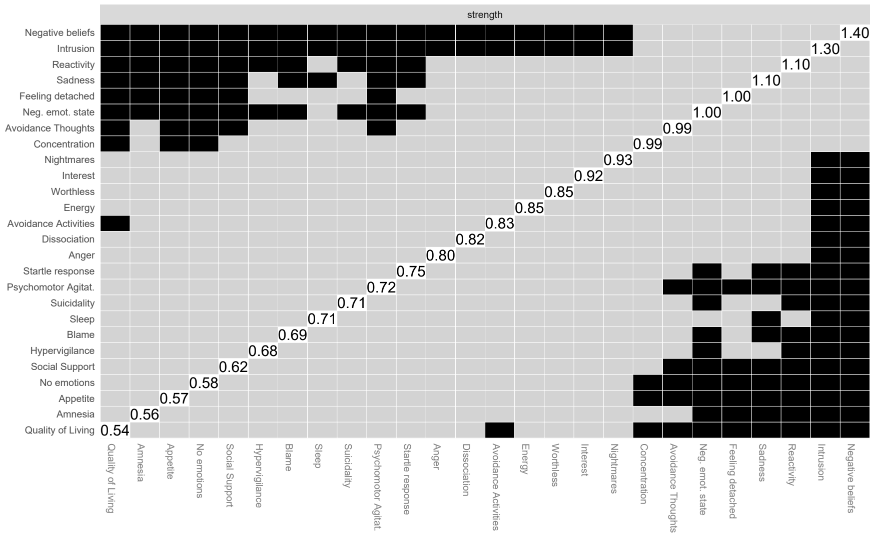

Fig. A7 Significant differences in node strength: Bootstrapped difference tests ($\alpha = .05$) between node strength of the 26 nodes with 2500 iterations. Black boxes indicate nodes with significant differences, gray boxes indicate nodes not differing significantly from one-another. Values in the white boxes represent node strength.
