# Supplementary figures and images for: A house is not a home: a network model perspective on the dynamics between subjective quality of living conditions, social support, and mental health of refugees and asylum seekers
Source: Soc Psychiatry Psychiatr Epidemiol. 2023 Jan 12;58(5):757–68. doi: 10.1007/s00127-022-02419-3 (PMC10097787; doi:10.1007/s00127-022-02419-3)

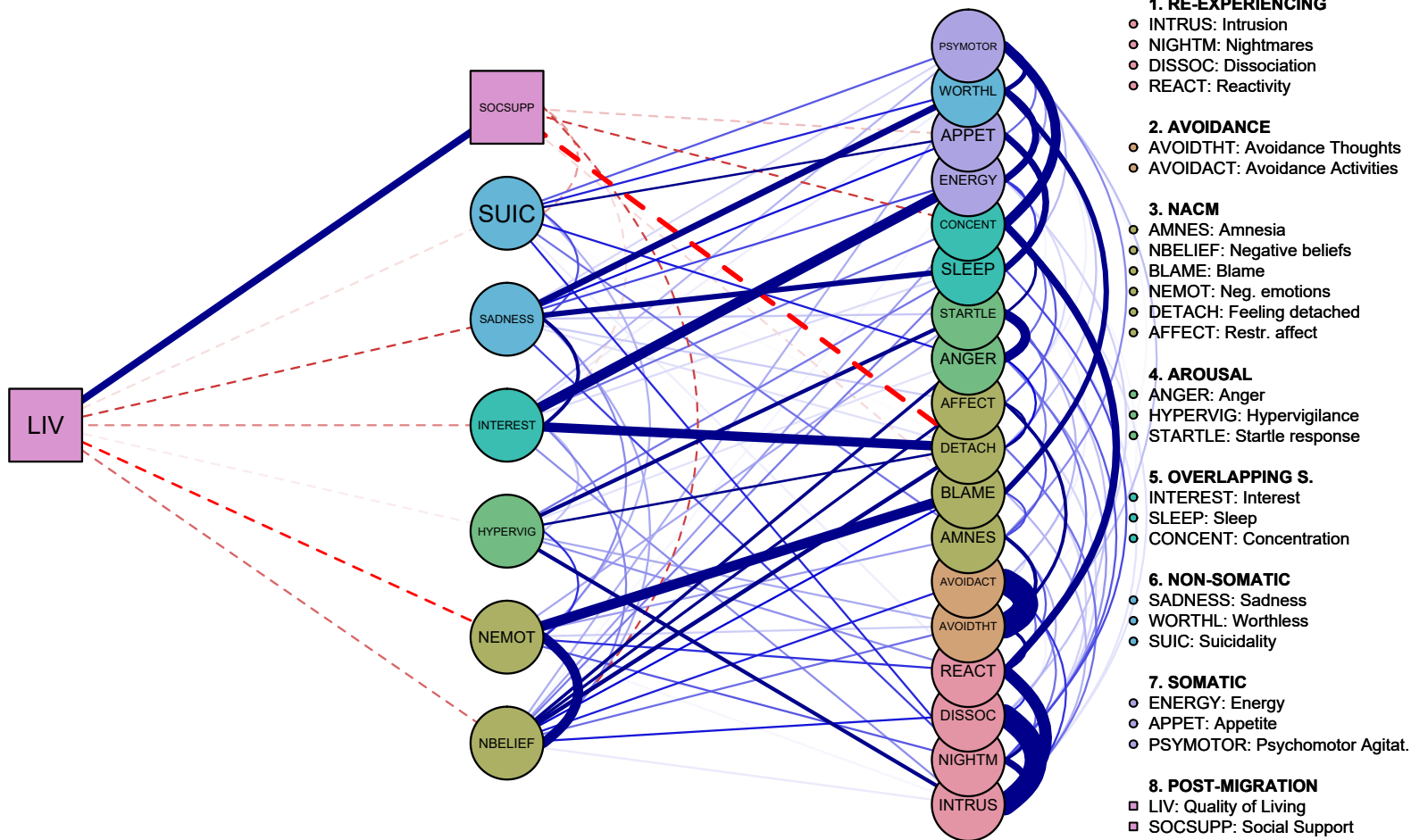

Supplement: Supplementary file 2 — (pdf 66 KB) [file 127_2022_2419_MOESM2_ESM.pdf]

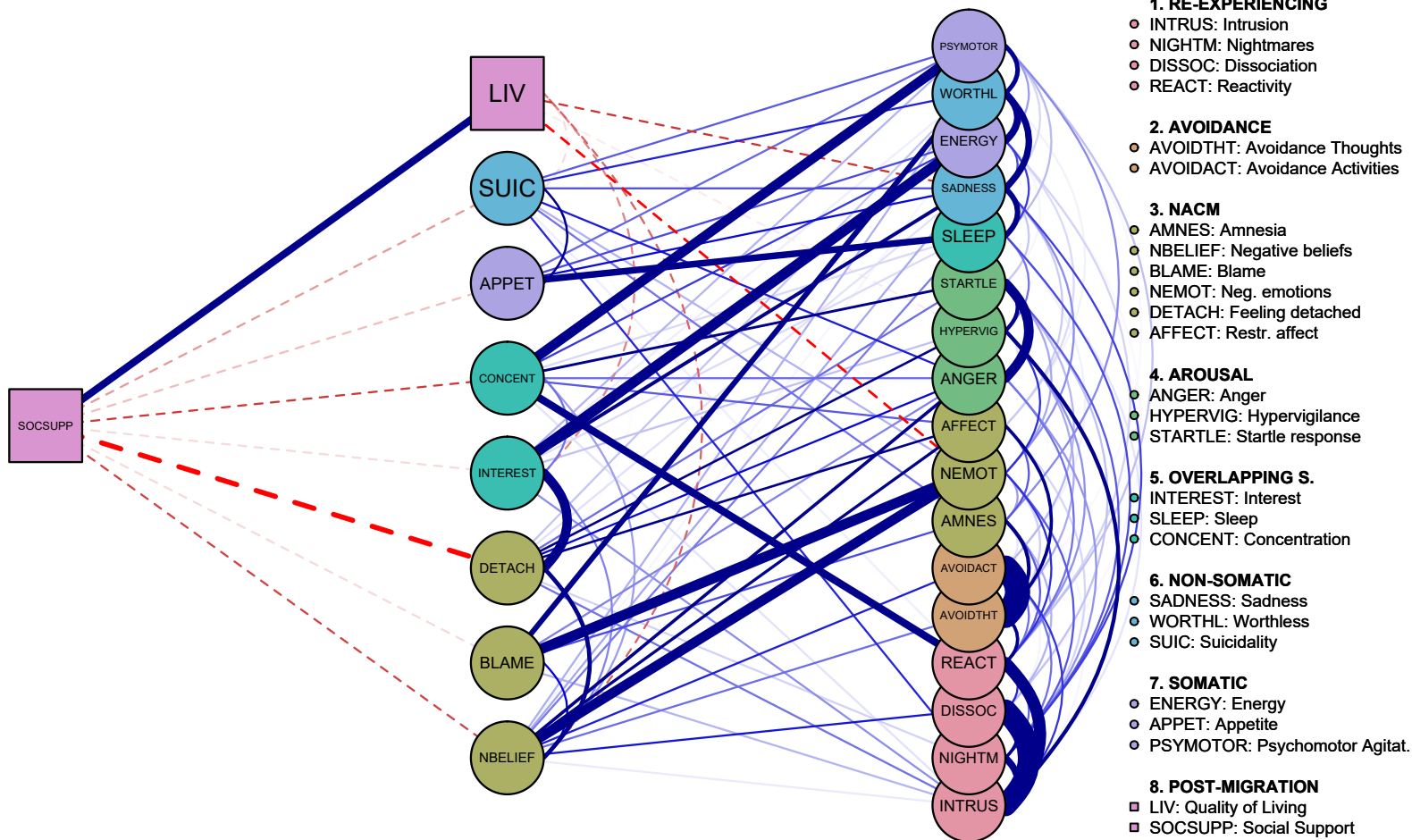

Supplement: Supplementary file 3 — (pdf 65 KB) [file 127_2022_2419_MOESM3_ESM.pdf]

edge

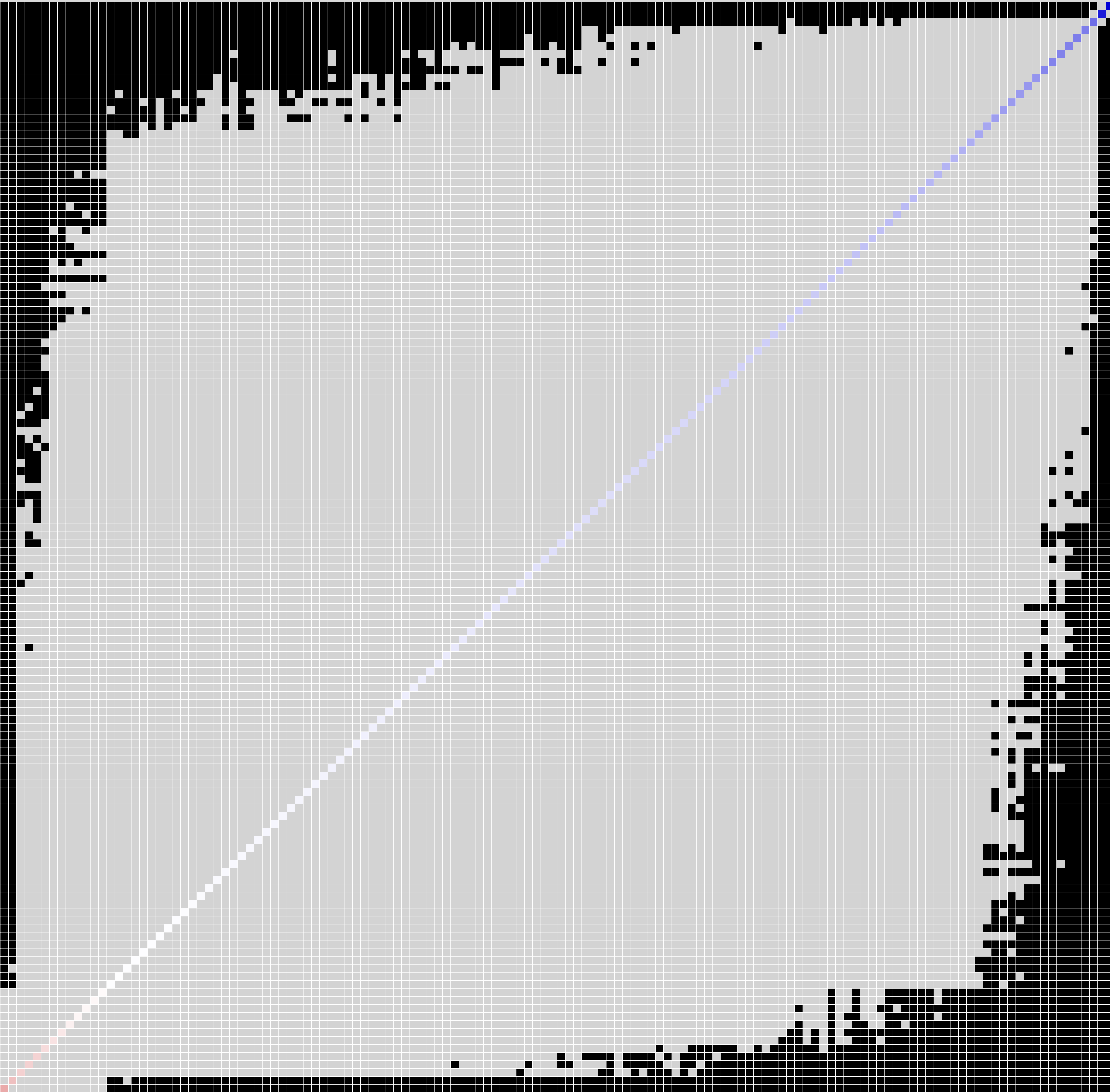

Supplement: Supplementary file 4 — (pdf 98 KB) [file 127_2022_2419_MOESM4_ESM.pdf]

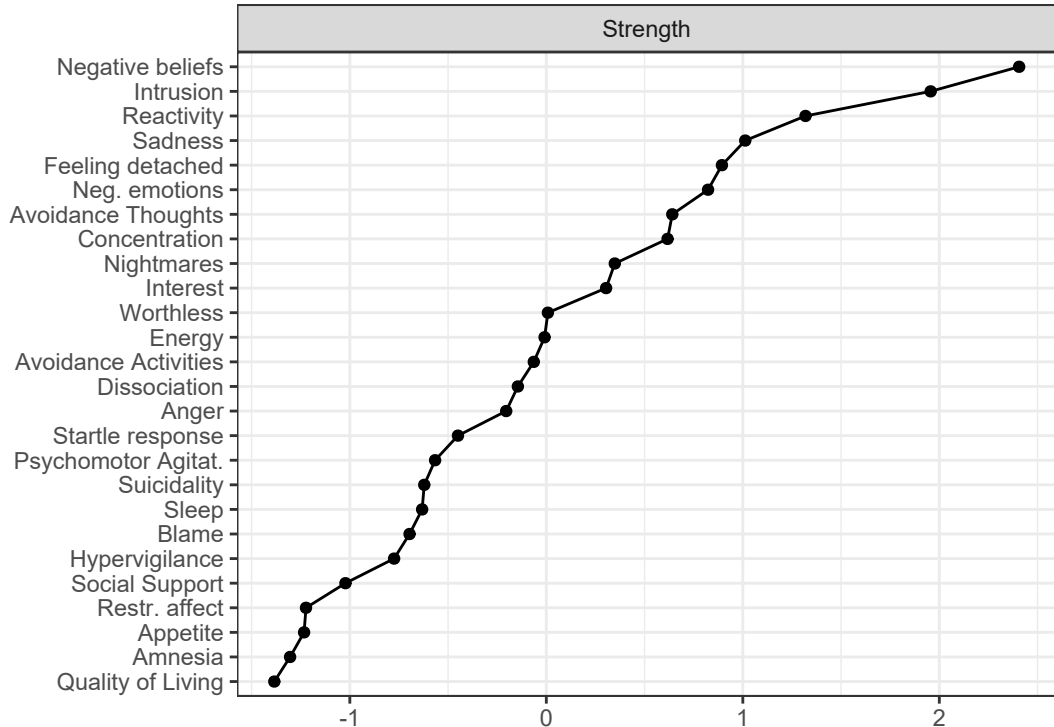

Supplement: Supplementary file 5 — (pdf 23 KB) [file 127_2022_2419_MOESM5_ESM.pdf]

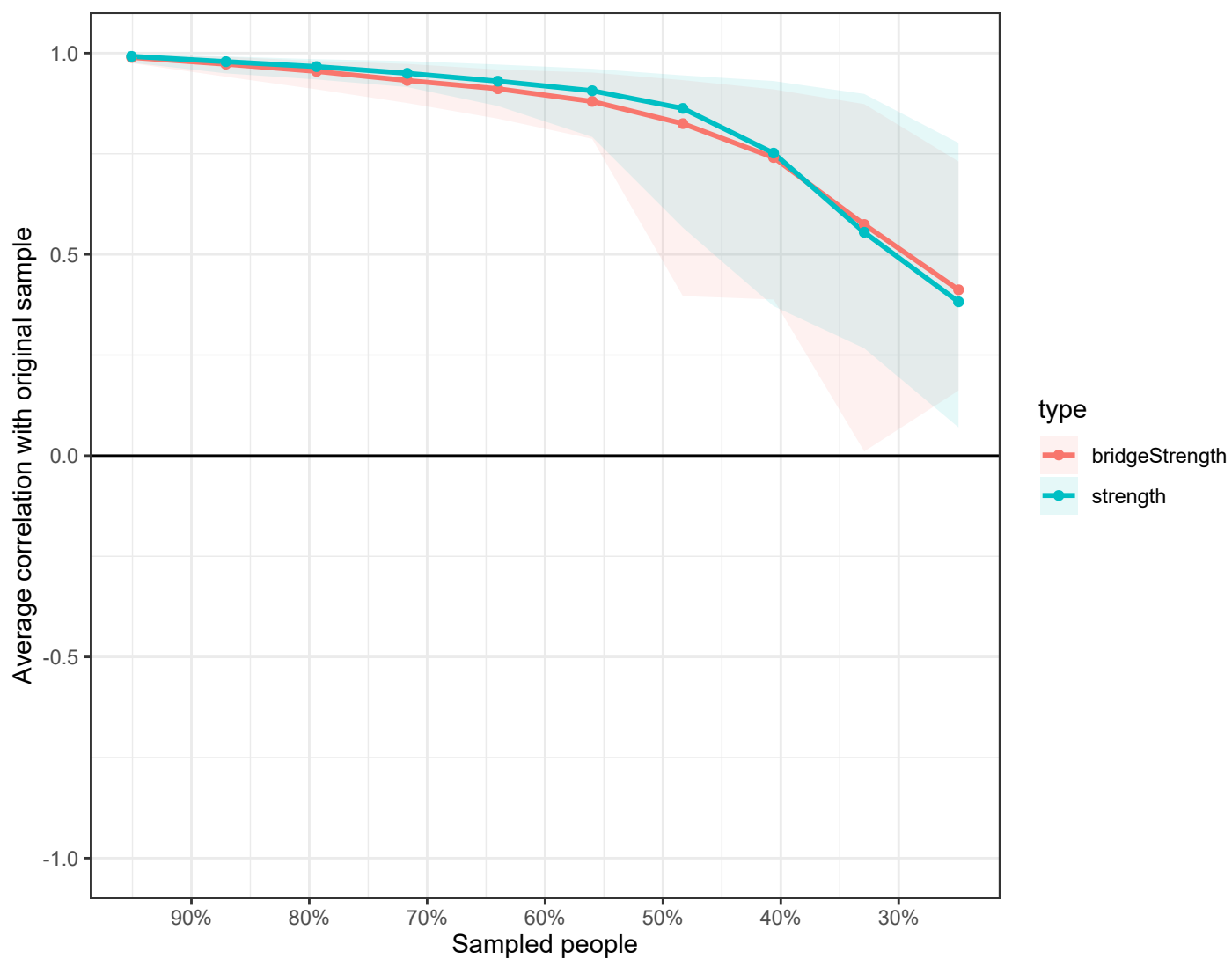

Supplement: Supplementary file 6 — (pdf 22 KB) [file 127_2022_2419_MOESM6_ESM.pdf]

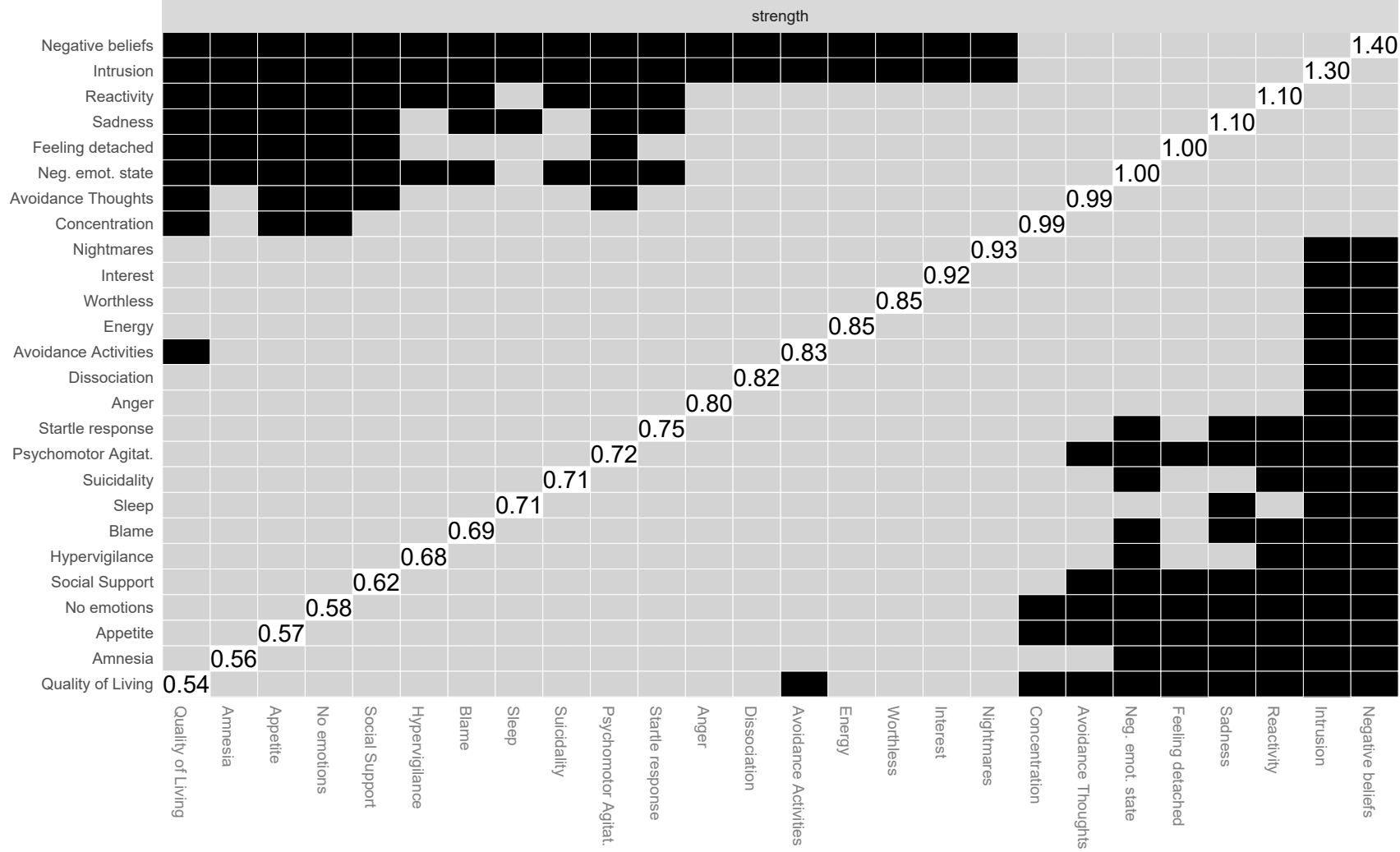

Supplement: Supplementary file 7 — (pdf 29 KB) [file 127_2022_2419_MOESM7_ESM.pdf]
